# Genetic Deletion of Laminin Isoforms β2 and γ3 Induces a Reduction in Kir4.1 and Aquaporin-4 Expression and Function in the Retina

**DOI:** 10.1371/journal.pone.0016106

**Published:** 2011-01-21

**Authors:** Petra G. Hirrlinger, Thomas Pannicke, Ulrike Winkler, Thomas Claudepierre, Shweta Varshney, Christine Schulze, Andreas Reichenbach, William J. Brunken, Johannes Hirrlinger

**Affiliations:** 1 Paul Flechsig Institute of Brain Research, University of Leipzig, Leipzig, Germany; 2 Carl Ludwig Institute of Physiology and Interdisciplinary Centre for Clinical Research, University of Leipzig, Leipzig, Germany; 3 Department of Ophthalmology and Eye Hospital, University of Leipzig, Leipzig, Germany; 4 Departments of Cell Biology and Ophthalmology, SUNY Downstate Medical Center, Brooklyn, New York, United States of America; Université de Technologie de Compiègne, France

## Abstract

**Background:**

Glial cells such as retinal Müller glial cells are involved in potassium ion and water homeostasis of the neural tissue. In these cells, inwardly rectifying potassium (Kir) channels and aquaporin-4 water channels play an important role in the process of spatial potassium buffering and water drainage. Moreover, Kir4.1 channels are involved in the maintenance of the negative Müller cell membrane potential. The subcellular distribution of Kir4.1 and aquaporin-4 channels appears to be maintained by interactions with extracellular and intracellular molecules. Laminins in the extracellular matrix, dystroglycan in the membrane, and dystrophins in the cytomatrix form a complex mediating the polarized expression of Kir4.1 and aquaporin-4 in Müller cells.

**Methodology/Principal Findings:**

The aim of the present study was to test the function of the β2 and γ3 containing laminins in murine Müller cells. We used knockout mice with genetic deletion of both β2 and γ3 laminin genes to assay the effects on Kir4.1 and aquaporin-4. We studied protein and mRNA expression by immunohistochemistry, Western Blot, and quantitative RT-PCR, respectively, and membrane currents of isolated cells by patch-clamp experiments. We found a down-regulation of mRNA and protein of Kir4.1 as well as of aquaporin-4 protein in laminin knockout mice. Moreover, Müller cells from laminin β2 and γ3 knockout mice had reduced Kir-mediated inward currents and their membrane potentials were more positive than those in age-matched wild-type mice.

**Conclusion:**

These findings demonstrate a strong impact of laminin β2 and γ3 subunits on the expression and function of both aquaporin-4 and Kir4.1, two important membrane proteins in Müller cells.

## Introduction

In the murine retina, Müller cells constitute the dominant macroglia. Müller cells are specialized radial glial cells which span the entire thickness of the retina. This radial shape provides the potential to be implicated in retinal development, function and integrity, and to exert many functions that require an intimate interaction with neurons and synapses [1]. Müller cells also contact both the vitreal chamber by enlarged basal end-feet and subretinal space (the remnant of the ventricular compartment) by apical microvillar specializations. Moreover, Müller cells are thought to participate in the induction, maintenance, and function of the blood retina barrier [1], [2]. In neural tissues, such as the retina, the maintenance of extracellular potassium ion homeostasis is a crucial function of glia cells [3]. Müller cells take up potassium ions released during neuronal activity, and redistribute them into the vascular, vitreal, and subretinal compartments [4], [5]. To buffer potassium these cells express a high density of specific inwardly rectifying potassium channels (Kir). Moreover, potassium fluxes are coupled to the water transport; this is accomplished by the parallel spatial distribution of water transport proteins, *viz.* the aquaporin-4 water pore [6], [7]. In healthy retinae these transport proteins are enriched in the vitreal endfeet and in cell processes directly contacting intraretinal blood vessels [7]. This specific localization is required for proper channel functioning. In the diseased retina of various species, a strong downregulation of Kir4.1 protein expression as well as a reduction of amplitudes of potassium currents have been shown [8]–[11]. In addition to their role in ion homeostasis, Kir4.1 channels are involved in the maintenance of the negative membrane potential of Müller cells as well as of other glial cells [4]. Thus, the functional expression of Kir4.1 channels in glial membranes is important for voltage-dependent transport processes, such as glutamate uptake [12], [13]. The important role of the Kir4.1 channels is demonstrated by a number of studies identifying mutations in the Kir4.1 gene (*KCNJ10*) as a reason for symptoms found in the EAST syndrome (epilepsy, ataxia, sensorineural deafness, and tubulopathy) [14]–[18]. Moreover, possible associations between polymorphisms of the gene for aquaporin-4 and epilepsy have been described [19].

Laminins are cell adhesion molecules found in the extracellular matrix (ECM) – predominantly, in basement membranes [20], [21] – such as in the inner limiting membrane (ILM), a specialized basement membrane separating the retina from the vitreous body. These glycoproteins serve as nucleating factors for the formation of basement membranes, and bind to a variety of cell surface receptors [22] that connect the proteins of the ECM to the cytoskeleton [23] via the dystrophin-associated protein (DAP) complex. Laminins are heterotrimers composed of α, β, and γ subunits [24]. The different laminin subunits are expressed tissue-specifically and appear to assemble into 18 proposed isoforms [22]. The β2 and the γ3 subunits have been shown to be coexpressed with different α-chains in the three neural laminins α3β2γ3, α4β2γ3 and α5β2γ3 (also known as laminins 13, 14, and 15, respectively), the last two have been found in the retina [25]–[27]. Laminins are vital for many physiological functions. It was demonstrated in Müller cell cultures that the presence of extracellular laminin is a prerequisite for the clustered expression of Kir4.1 [28]. Furthermore, disruptions in laminin expression or deposition are implicated in several human diseases of the central nervous system including the retina [22].

A mutation in the laminin β2 subunit gene has been shown to cause the Pierson syndrome, a severe congenital disorder with early lethality [29]. Patients show, in addition to other defects, a loss of vision. Mice deficient in the β2 subunit begin to die around postnatal day 21–28, show deficits in neuromuscular synapses [30] and kidney glomeruli [31], and a retinal pathology [32]–[34]. Whereas single deletion of either β2 or γ3 causes only small and almost no effects on retinal morphology, respectively, the double mutation of both genes results in severe defects in the formation of the ILM which leads to retinal dysplasia [33], [34].

To investigate the effect of the loss of both laminin β2 and γ3 subunits on functional expression of Kir4.1 and aquaporin-4 of retinal Müller cells, mice with a genetic deletion of both subunits were used in this study. Here we show that double knockout animals exhibit a reduced content of Kir4.1 and aquaporin-4 protein and Kir4.1 mRNA level, as well as reduced amplitudes of Kir-mediated currents and less negative membrane potentials.

## Materials and Methods

### Animals and Ethics statement

Mice devoid of laminin β2 (NM_008483.3) and γ3 (NM_011836.3) (laminin subunit double knockouts - β2^−/−^/γ3^−/−^, [34]) of both sexes (C57/Bl6 background) were used. Mouse breeding was performed in the animal facilities of the Faculty of Medicine, University of Leipzig according to European (Council Directive 86/609/EEC) and German (Tierschutzgesetz) guidelines for the welfare of experimental animals and were approved by the local authorities (Landesdirection Leipzig, permit number T32/10). Mice were housed in a 12 h/12 h light dark cycle with access to food and water ad libitum. Double knockout animals start to die at postnatal day 21. Therefore only 3–4 weeks old animals were used in this study.

### Immunohistochemistry

Eyes of three β2^−/−^/γ3^−/−^and three wild-type animals were fixed in 4% paraformaldehyde in phosphate-buffered saline (PBS) overnight. For immunostaining of retinal slices isolated retinal tissues were embedded in PBS containing 3% agarose and cut into 40 µm thick slices using a vibratome. The slices or retinal wholemounts were permeabilized with 0.4% Triton X-100 in PBS for 30 minutes, blocked in 4% goat serum/0.2% Triton for 30 minutes, and incubated overnight with the primary antibody in 1% goat serum/0.05% Triton. The secondary antibody was applied for two hours in 1.5% goat serum in PBS. The following antibodies were used: rabbit anti Kir4.1 (Sigma-Aldrich, Taufkirchen, Germany 1∶100), rabbit anti aquaporin-4 (Sigma, 1∶200), rabbit anti vimentin (Biomeda V2009, 1∶100), rabbit anti laminin (“pan-laminin“, Sigma-Aldrich, 1∶200), rabbit anti GFAP (DAKO, Hamburg, Germany, 1∶500), mouse anti utrophin (Novocastra, Lab. Ltd, Newcastle, UK, 1∶4), goat anti rabbit-Cy3, and goat anti mouse-Cy3 (both Dianova, Hamburg, Germany, 1∶1000).

For immunocytochemical staining of isolated cells the retina obtained from three β2^−/−^/γ3^−/−^ and three wild-type mice were incubated in PBS containing 0.3 mg/ml papain (Roche, Mannheim, Germany) for 30 minutes at 37°C, followed by washing with PBS. After short incubation with DNase I (200 U/ml; Sigma-Aldrich) the tissue was fixed in 4% PFA for 20 min, washed three times in PBS and afterwards dissociated. Isolated Müller cells were identified morphologically and stored at 4°C in PBS/0.001% sodium azide. Cells were plated on Superfrost slides and allowed to adhere. The Müller cell endfoot could be unequivocally identified using differential interference contrast microscopy. The cells were incubated in PBS/0.1% glycine for 10 min, permeabilized for 10 min with PBS/0.1% glycine/0.3% Triton X-100, and incubated for 2 h with the primary antibodies in PBS/10% goat serum. After washing the secondary antibodies were applied for 30 min. The following antibodies were used: rabbit anti Kir4.1 (Sigma-Aldrich, 1∶100), rabbit anti aquaporin-4 (Sigma-Aldrich, 1∶200), mouse anti glutamine synthetase (Millipore/Chemicon, Schwalbach/Ts, Germany 1∶500), goat anti mouse-Cy2 (Dianova, 1∶100), goat anti rabbit-Cy3 (Dianova, 1∶1000). Omitting primary antibodies demonstrated lack of unspecific labelling.

Three dimensional image stacks with x-y frame sizes of 1024×1024 pixels (pixel size 0.29×0.29 µm, isolated cells) and 2048×2048 (pixel size 0.15×0.15 µm, retinal slices) at intervals of 1 µm in the z-direction were obtained using a confocal laser scanning microscope (Zeiss LSM 510NLO; Axioskop FS2M, Zeiss, Oberkochen, Germany) and a C-Apochromat 40×/1.2 water immersion objective. Image stacks were stored, converted to maximum intensity projections and filtered using median filter with the Zeiss LSM software and Adobe Photoshop.

For quantification of the expression of Kir4.1 and aquaporin-4 in isolated Müller cells double immunostainings for glutamine synthetase and Kir4.1 or aquaporin-4 were used. Within the original image stacks obtained by confocal microscopy as described above, voxels inside a cell were identified by thresholding the glutamine synthetase staining, which labels the whole Müller cell. Then, the mean grey intensity of the Kir4.1 or aquaporin-4 staining of all voxels belonging to this cell was calculated. At least a minimum of 22 cells derived from minimal 3 mice have been analyzed. Values are given as mean ± sem in % of the control value (wild-type) for the whole cell and for three different parts of it: endfoot, middle part (including soma and inner process), and distal end. Mann-Whitney U test was used for statistical analysis Care was taken to stain control and β2^−/−^/γ3^−/−^ cells in parallel using the same solutions as well as to acquire the confocal images with exactly the same settings of the microscope.

### Western Blot

Retinae of three β2^−/−^/γ3^−/−^ animals and three littermate controls were homogenized in lysis buffer A (50 mM Tris-HCl, pH 7.4, 150 mM NaCl, 1 mM EDTA, 1x Complete Protease Inhibitor cocktail [Roche]). Homogenates were centrifuged at 13,000 rpm for 15 min at 4°C. The resulting pellet was solubilized in 50 µl of lysis buffer B (50 mM Tris-HCl, pH 7.4, 150 mM NaCl, 1 mM EDTA, 1x Complete Protease Inhibitor cocktail (Roche), 1% Triton X-100, 0.1% SDS). 5 µg (GAPDH), 8 µg (aquaporin-4) or 10 µg (Kir4.1) of protein of each sample was run on a 12% SDS-polyacrylamide gel and blotted on nitrocellulose membrane (Amersham, Freiburg, Germany). After blocking with 5% milk powder and 10% Roti-Block (Roth, Karlsruhe, Germany) overnight at 4°C the membranes were probed with primary antibodies (rabbit anti Kir4.1 1∶500, Sigma-Aldrich; rabbit anti aquaporin-4, 1∶400, Sigma-Aldrich; mouse anti GAPDH 1∶5000, Ambion, Austin, Texas) in 5% milk powder for 1 h at room temperature. After washing, membranes were incubated with secondary antibodies (HRP goat anti rabbit 1∶1000, HRP goat anti mouse 1∶1000, Jackson Immuno Research, Newmarket, UK) in 5% milk powder for 30 min at room temperature and washed again. Signals were developed with ECL solution (0.65 mM luminol, 2.7 mM H_2_O_2_, 0.5 mM p-hydroxycoumarine acid) and detected using a G-Box imager (SynGene, Cambridge, UK). Using fluorescently labelled secondary antibodies and detection by an Odyssey scanner (Licor Bioscience, Lincoln, NE, USA) the down regulation of aquaporin-4 and Kir4.1 expression in β2^−/−^/γ3^−/−^ animals was confirmed (data not shown).

### Patch-clamp recordings

Retinal pieces were incubated in Ca^2+^/Mg^2+^ free PBS (Biochrom, Berlin, Germany) containing 0.3 mg/ml papain (Roche) for 30 minutes at 37°C, followed by washing with PBS. After short incubation with DNase I (200 U/ml; Sigma-Aldrich) the tissue was dissociated mechanically using a 1-ml pipette tip. Isolated Müller cells were stored at 4°C in minimum essential medium (Sigma-Aldrich) until use within three hours and could be identified morphologically. Electrophysiological recordings were performed in the whole-cell configuration of the patch-clamp technique at 20-24°C using the patch-clamp amplifier Axopatch 200A (Axon Instruments, Foster City, CA, USA). Cells were suspended in extracellular solution in a chamber on the stage of a microscope (Axioskop, Zeiss, Germany). Extracellular solution contained (mM): NaCl, 135; KCl, 3; CaCl_2_, 2; MgCl_2_, 1; Na_2_HPO_4_, 1; glucose, 11; N-(2-hydroxyethyl)piperazine-N'-2-ethanesulfonic acid (HEPES)-Tris, 10; pH 7.4. The chamber was continuously perfused (2 ml/min) after establishing the whole-cell configuration. Recording electrodes from borosilicate glass had resistances of 5 MΩ when filled with a solution containing (mM): KCl, 130; NaCl, 10; MgCl_2_, 2; CaCl_2_, 1; ethylene glycol-bis(2-aminoethylether)-N,N,N',N'-tetraacetic acid (EGTA), 10; HEPES-Tris, 10; pH 7.1. Currents were low-pass filtered at 1 kHz and digitized at 5 kHz using a 12-bit A/D converter. Voltage command protocols were generated and data analysis was performed with the software ISO 2 (MFK, Niedernhausen, Germany). To evoke membrane currents, de- and hyperpolarizing voltage steps of 250 ms duration were applied from a holding potential of −80 mV. The steady-state currents were measured at the end of the steps, and the amplitude of the inward currents was recorded at −140 mV. The membrane capacitance of the cells was calculated from the integral of the uncompensated capacitive artefact. Membrane potentials were recorded in the current-clamp mode at I = 0. Mean values with standard deviations are given. Student's t-test was used for statistical analysis.

### Quantitative RT-PCR

The retina of 7 β2^−/−^/γ3^−/−^ and 9 wild-type control mice (age: 17–23 days) was isolated and RNA was prepared using the RNeasy mini kit (Qiagen, Hilden, Germany) according to the manufacturer's instructions. RNA was reverse transcribed using Superscript III reverse transcriptase (Invitrogen, Darmstadt Germany) and random hexamer primers. qPCR was performed on a AB7300 real-time PCR cycler (Applied Biosystems Invitrogen, Darmstadt Germany) using the SYBR Green ROX Absolute qPCR Mastermix (Thermo Fisher Scientific, Darmstadt, Germany). The following primer pairs were used: Kir4.1: 5′-AGTCTTGGCCCTGCCTGT-3′ and 5′-AGCGACCGACGTCATCTT-3′; aquaporin-4: 5′-TGGAGGATTGGGAGTCACC-3′ and 5′-TGAACACCAACTGGAAAGTGA-3′; GFAP: 5′-ACAGACTTTCTCCAACCTCCAG-3′ and 5′-CCTTCTGACACGGATTTGGT-3′; GAPDH: 5′-TGTCCGTCGTGGATCTGAC-3′ and 5′-CCTGCTTCACCACCTTCTTG-3′; β-actin: 5′-CTTCCTCCCTGGAGAAGAGC-3′ and 5′-ATGCCACAGGATTCCATACC-3′. The data shown has been normalized to the expression of GAPDH using the ΔC_t_-method and are given as mean ± sem; the expression level in wild-type animals has been set as 1. Similar results have been obtained after normalization on actin expression (data not shown).

## Results

### Histochemical data

**Figure 1:**
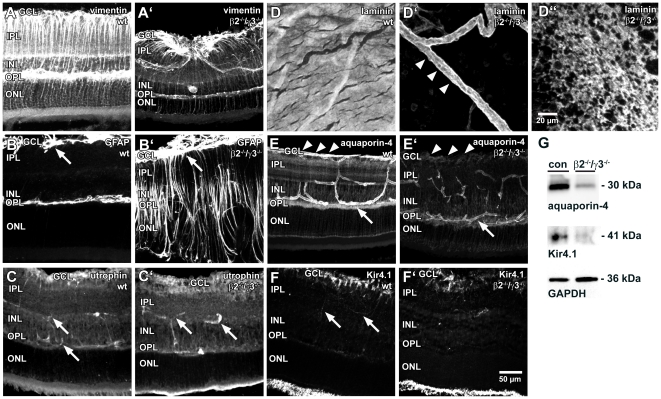
Immunohistochemical analysis of retinae of β2, γ3 laminin knockout mice. Immunohistochemical stainings of fixed retinal slices (A–C, E–F) or wholemounts (D) from wild-type (wt, A–F) and β2, γ3 laminin knockout mice (β2^−/−-^/γ3^−/−^, A′–F′, D″) are shown. A, A′: Staining for the Müller cell filament protein vimentin revealed a remarkable disorganisation of the Müller cell orientation in certain parts of the retina from β2^−/−^/γ3^−/−^ mice. B, B′: Immunostaining for GFAP, a marker for reactive gliosis, indicates a strong upregulation in Müller cells of β2^−/−^/γ3^−/−^ mice. In the wild-type retina, retinal astrocytes located in the ganglion cell layer (GCL) express GFAP (arrow in B). In the knockout animal, not only glial elements in the GCL (astrocytes and Müller cell endfeet, arrow in B′) are GFAP-positive, but also Müller cells spanning all retinal layers (B′). C,C′: An immunostaining against utrophin which may connect channels to the DAP complex did not reveal remarkable differences between wild-type and knockout animals. Arrows point to intraretinal blood vessels which are contacted by immunopositive glial membranes. D-D″: The inner limiting membrane could be stained with a pan-laminin antibody at the vitreal side of wholemount preparations from wild-type animals (D). This staining was lacking in some parts of the β2^−/−^/γ3^−/−^ retina (D′). Arrowheads in D′ mark a laminin-positive blood vessel. In regions where the inner limiting membrane still existed, it displayed a netlike structure (D″). E, E′: The aquaporin-4 located at retinal blood vessels was similar in both groups (arrows mark a blood vessel in the outer plexiform layer (OPL). Whereas Müller cell endfeet contacting the inner limiting membrane (arrowheads) were aquaporin-4-positive in the wild-type retina, this staining could not be observed in β2^−/−^/γ3^−/−^ mice. F, F′: Prominent Kir4.1 immunostaining was found in Müller cell endfeet in the GCL. This staining was found to be reduced in the knockout situation. Weak immunostaining was found in structures likely to be Müller cell processes in the inner plexiform layer (IPL, arrows in F). Photoreceptor segments displayed strong autofluorescence (lower edge of the images). G: Western blot analysis of aquaporin-4 (30 kDa), Kir4.1 (41 kDa), and GAPDH (36 kDa) as loading control in retinal homogenates of littermate controls (con, left lanes) and of β2^−/−^/γ3^−/−^animals (β2^−/−^/γ3^−/−^, right lanes). Protein expression of both channel proteins is lower in β2^−/−^/γ3^−/−^ animals compared to controls. Scale bar in F′ applies to all panels except D″. Retinal slices in A-C and E-F are shown with the vitreal side upwards. INL, inner nuclear layer, ONL, outer nuclear layer.

Prior studies of the retina of laminin mutant mice have shown a variety of defects, including disruptions in the photoreceptor synapse [32], the development of the dopaminergic system [33] and structural organization of retinal lamination [34]. In each of these studies, disruptions in the organization of the Müller cell have been noted. In this study, we extend these initial observations and specifically investigate how Müller cell structure and function are compromised in the laminin knockout mice focusing on the phenotype of the laminin β2^−/−^/γ3^−/−^ mouse line. To investigate the morphology of Müller cells, we used an antibody against the Müller cell specific filament protein vimentin. In wild-type retinae, Müller cells span the tissue in a perpendicular course (Fig. 1A). In most regions of the β2^−/−^/γ3^−/−^ retina Müller cells showed a similar morphology, some Müller cells were found with profoundly altered trajectories; that is their vitreally directed processes were bent and appeared to converge towards a single point in the retina containing perhaps intact parts of the ILM (Fig. 1A′). By using an antibody against GFAP, a marker for reactive gliosis in Müller cells, we could clearly demonstrate a dramatic increase in GFAP expression in β2^−/−^/γ3^−/−^ mice (Fig. 1B′) indicating that Müller cells were undergoing gliosis, a pathobiologic response, in β2^−/−^/γ3^−/−^ retina. Previous studies suggested that laminin deletion disrupted the organization of the ILM [34]. We used a pan-laminin antibody and stained retinal wholemounts to examine the ILM. Wild-type retinae displayed a homogeneous staining, demonstrating a continuous layer (Fig. 1D), while in β2^−/−^/γ3^−/−^ retinae the ILM was lacking in most parts of the retina; only the blood vessels were laminin positive (Fig. 1D′). Importantly, in areas with remaining ILM, the ILM showed a discontinuous, netlike pattern (Fig. 1D″). Thus, our initial experiments confirmed those prior studies.

As has been shown before [35]–[37], Müller cells contain proteins of the DAP-utrophin complex. To investigate whether the intracellular part of this complex is altered in β2^−/−^/γ3^−/−^ mice, we immunostained retinal slices with antibodies against utrophin. No remarkable differences were found, demonstrating that lack of β2 and γ3 laminins does not affect the expression and localization of this cytoskeletal protein (Fig. 1C,C′).

We proceeded to analyse the Müller cell organization by studying the expression of Kir4.1 and aquaporin-4 proteins in the retina of β2^−/−^/γ3^−/−^ and wild-type mice (Fig. 1E,F). Aquaporin-4 in the wild-type retina is mainly localized around blood vessels and at the ILM, whereas a weak staining was found in the inner and outer plexiform layers (Fig. 1E). In retinae from β2^−/−^/γ3^−/−^ mice, the staining was qualitatively similar but several disruptions in the spatial localization of aquaporin-4 were noted. Whereas blood vessels were clearly stained in the knockout retina, a distinct immunolabeling of the ILM was lacking (Fig. 1E′). Kir4.1 immunostaining in the wild-type animal was mainly located to the endfeet of Müller cells at the vitreal border of the retina. This apparent concentration of Kir4.1 channel protein in the ganglion cell layer (GCL) was considerably reduced in β2^−/−^/γ3^−/−^ mice (Fig. 1F,F′). Moreover, both aquaporin-4 and Kir4.1 stainings of the knockout retina (Fig. 1E′,F′) showed morphological alterations and a disturbed retinal layering. These results suggest that there is a redistribution of aquaporin-4 and Kir4.1 channels in the retinal architecture as well as a reduction in their expression levels. The reduction of aquaporin-4 and Kir4.1 protein expression in the retina was confirmed by Western blot analysis of retinal homogenates (Fig. 1G).

**Figure 2:**
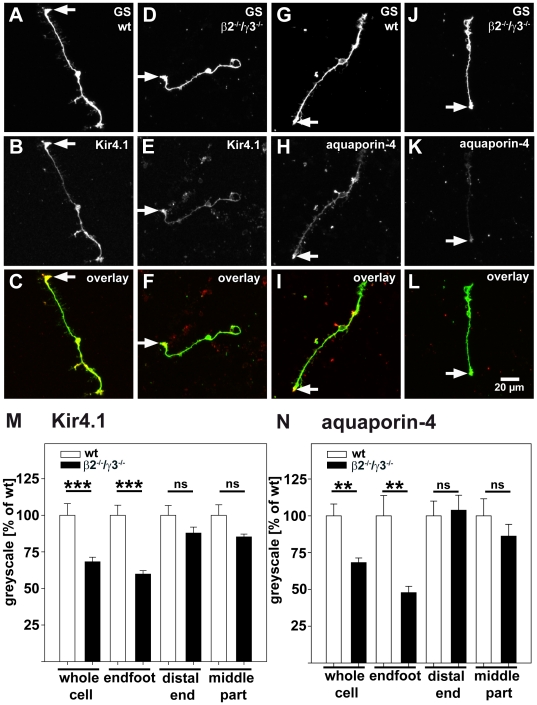
Immunocytochemical staining of acutely isolated Müller cells from β2, γ3 laminin knockout mice. β2, γ3 laminin knockout cells (β2^−/−^/γ3^−/−^) are shown in panels D–F and J–L; wild-type controls in A–C and G–I. The expression of Kir4.1 (B, E) and aquaporin-4 (H, K) was analysed in glutamine synthetase (GS)-positive Müller cells (A, D, G, J). C, F, I, L show overlays of GS (green) and Kir4.1 (red, C, F) or aquaporin-4 (red, I, L) staining, respectively. Endfeet of the cells are marked by arrows. The staining was quantified as described in the methods. In Müller cells from β2^−/−^/γ3^−/−^ retinae the overall level of Kir4.1 staining (M) and of aquaporin-4 staining (N) quantified in whole cells was significantly lower as compared to wild-type Müller cells wild-types (wild-type: white bars; β2^−/−^/γ3^−/−^: black bars). These differences are mainly due to highly significant changes in endfeet expression levels of both proteins (M, N). Numbers of cells analysed was between 22 and 28 for wild-types and between 30 and 66 for β2^−/−^/γ3^−/−^ mice; *** P≤0.001, ** P<0.01.

As both Kir4.1 and aquaporin-4 are expressed heavily in Müller cells, we next characterized the expression of Kir4.1 and aquaporin-4 in more detail within the Müller cells themselves. Müller cells were isolated from the retinae and unambiguously identified by staining against glutamine synthetase, which essentially labeled all parts of the Müller cells from both the wild-type and β2^−/−^/γ3^−/−^ mice (Fig. 2). Kir4.1 staining was found in cells from wild-type as well as β2^−/−^/γ3^−/−^ mice and was concentrated in the endfeet of these cells. The staining in the β2^−/−^/γ3^−/−^ cells was weaker throughout the cell (Fig. 2B,E). Immunostaining against aquaporin-4 displayed similar alterations (Fig. 2H,K). Immunoreactivity of both channels in Müller cells from β2^−/−^/γ3^−/−^ was reduced throughout the cells.

To confirm this qualitative analysis, quantitative assessments of the expression levels of aquaporin-4 and Kir 4.1 were made from acutely isolated Müller cells. Müller cells from wild-type and β2^−/−^/γ3^−/−^ mice were stained in parallel, using the same solutions, and images were acquired using identical settings of the confocal laser scanning microscope. With this method the expression levels of Kir4.1 and aquaporin-4 were quantified in glutamine synthetase positive Müller cells. In Müller cells from β2^−/−^/γ3^−/−^ retinae the levels of Kir4.1 and aquaporin-4 immunostaining were significantly lower than in wild-type Müller cells (Fig. 2M,N). To further investigate whether certain regions of the Müller cells were differentially affected, we quantified the immunostaining in the following separate parts of the Müller cells: endfoot, middle part and distal end. Significantly reduced staining for Kir4.1 and aquaporin-4 was found mainly in Müller cell endfeet, whereas alterations in the other parts of the cells were not significant (Fig. 2M,N). These data suggest that alterations in the endfoot of Müller cells are the reason for the reduced staining intensity in the isolated cells and this quantitative assessment is consistent with the anatomical redistribution *in situ*.

### Quantitative RT-PCR

**Figure 3:**
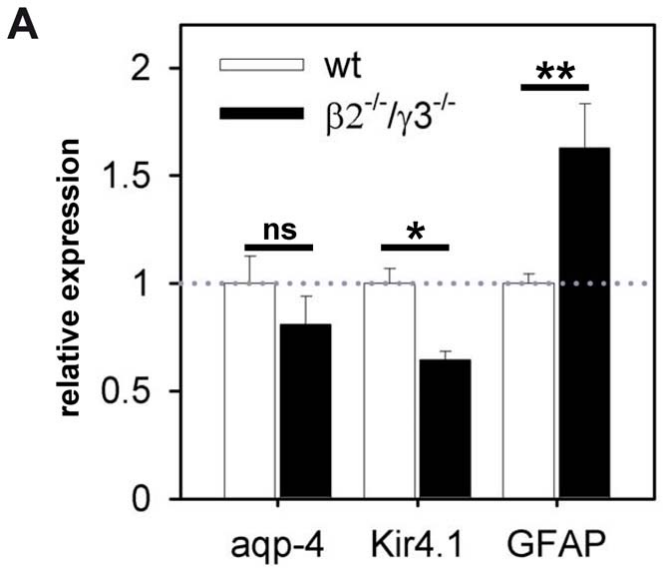
Relative expression of Kir4.1, aquaporin-4 and GFAP mRNA in wild-type and β2^−/−^/γ3^−/−^ retina. Values are normalized to GAPDH expression and were set as 1 for wild-types (n = 9 animals; white bars). In retinal tissue of β2^−/−^/γ3^−/−^ (n = 7 animals; black bars) the expression of Kir4.1 mRNA is significantly reduced (* P<0.05), while aquaporin-4 mRNA only shows a non-significant tendency to lower levels (ns: not significant; P = 0.597). In contrast, the expression level of GFAP, a marker for retinal gliosis, is upregulated (**P<0.01).

To analyse whether the decrease in protein expression of Kir4.1 and aquaporin-4 as shown by immunocytochemistry and Western Blot is reflected by a decrease in mRNA levels, RNA was harvested from wild-type and β2^−/−^/γ3^−/−^ retinae and analysed for mRNA levels using quantitative real time PCR (Fig. 3). Kir4.1 mRNA was significantly decreased (by 36%) in β2^−/−^/γ3^−/−^ mice as compared to control. In contrast, the mRNA level of aquaporin-4 was only slightly and not significantly reduced in the double knockout mice. Moreover, the expression of GFAP mRNA was also analysed. An increase in GFAP expression is an indicator for a gliotic response and has been documented in many types of retinal injuries or diseases [11], [38], [39]. Indeed, the expression of GFAP mRNA was found to be increased (Fig. 3) confirming the immunohistological staining in Fig. 1D.

### Electrophysiology

**Figure 4:**
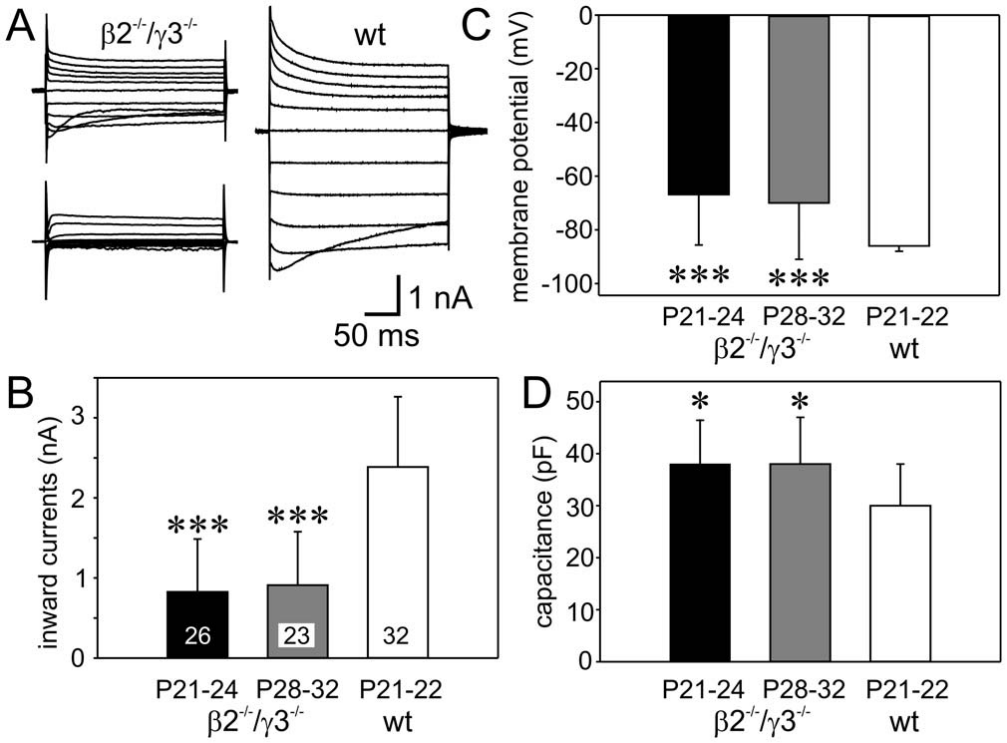
Electrophysiological properties of isolated Müller cells of mice at the age of 3–4 weeks. A: Depolarizing and hyperpolarizing voltage steps were applied from a holding potential of −80 mV with 20 mV increment. Whereas the current pattern of cells from wild-type mice (wt, right) was very similar to that shown before for adult mice, the currents recorded in cells from β2^−/−^/γ3^−/−^ mice had much smaller amplitudes (left). Moreover, the current patterns from β2^−/−^/γ3^−/−^ mice displayed a variability and inward currents were almost missing in some cells. Recordings were from a P21 (left, above), a P32 (left, below), and a P22 mouse (right) in β2^−/−^/γ3^−/−^ and wild-type animals, respectively. B–D: Significant alterations were observed in the inward currents at 60 mV hyperpolarization, the membrane potential and the membrane capacitance. Mean values with standard deviations are shown, numbers of recorded cells are given within the columns in B. Significant differences as compared to the wild-type control: *P<0.05, ***P<0.001.

Isolated Müller cells were used to investigate whether the deletion of laminin subunits β2 and γ3 had caused alterations of electrophysiological properties. β2^−/−^/γ3^−/−^ mice died in most cases at the age of four weeks or earlier. It has been reported for other mouse strains that the electrophysiological properties of Müller cells undergo remarkable alterations during the first 3 weeks of postnatal development [40], [41]. Therefore, we used Müller cells from wild-type (C57Bl/6) mice at the age of postnatal days 21 and 22 (P21/22) for control recordings (n = 32 cells from 5 animals). A typical example of the current responses to depolarizing and hyperpolarizing voltage steps and the mean values of certain electrophysiological properties are shown in Fig. 4. Most data are not significantly different to those from adult C57Bl/6 mice known from earlier studies [11], [42]. We therefore conclude that membrane currents have developed to maturity by the third postnatal week in wild-type mice. In contrast, we found that membrane currents failed to develop to maturity with the same temporal sequence in the β2^−/−^/γ3^−/−^ mice. The cells from mutant animals were pooled into two groups, depending on their age: P21-24 (n = 26 cells from 3 animals) and P28-32 (n = 23 cells from 4 animals). Although there was a considerable variability of the current patterns of Müller cells from β2^−/−^/γ3^−/−^ mice (Fig. 4A), it was evident that outward and inward currents at all recorded membrane potentials were clearly smaller than in control cells. Inward currents evoked by a 60-mV hyperpolarizing step - known to be mediated by Kir4.1 channels - were significantly reduced in both groups of β2^−/−^/γ3^−/−^ mice (Fig. 4B). The membrane potential was found to be less negative in β2^−/−^/γ3^−/−^ mice (Fig. 4C) consistent with the observation that the very negative membrane potential of Müller cells depends on the dominant Kir conductance [43], [44]. Finally, the membrane capacitance of Müller cells was slightly increased in β2^−/−^/γ3^−/−^ mice indicating that these cells have a larger membrane area (Fig. 4D) – a sign for cellular hypertrophy as typical for reactive glial cells [45].

## Discussion

In this study we used β2 and γ3 double knockout mice to investigate the impact of these laminin subunits on the functional expression of Kir4.1 and aquaporin-4 channels in Müller cells. It has been shown before in cultured Müller cells that extracellular laminin is necessary for a clustered expression of Kir4.1 in the Müller cell membrane [28], however, data from Müller cell cultures might be affected by the significant morphological alterations occuring *in vitro*. Müller cells are highly polarized cells with distinct subcellular compartments; their basal endfeet contact the ILM in order to maintain the retinal architecture and to achieve ionic exchange, their apical microvilli form adherens junctions with the photoreceptors at the outer limiting membrane towards the subretinal space; and lateral processes ensheath synapses and contact retinal blood vessels. In the retina of β2^−/−^/γ3^−/−^ mice, the integrity of the ILM is strongly disrupted [32], [34] as can be visualized by immunostaining with a pan-laminin antibody. Parts of the ILM are lacking, and the residual ILM has a netlike structure. This disruption is accompanied by a deterioration of Müller cell end feet morphology [34] and the loss of β-dystroglycan at Müller cell endfeet [34], [35]. β-dystroglycan is the transmembrane portion of the laminin receptor dystroglycan, a member of the DAP complex [36], [46] which anchors Kir4.1 and aquaporin-4 in Müller cells in a laminin-dependent manner. Kir4.1 and aquaporin-4 as well as β-dystroglycan and dystrophins are concentrated in the endfeet of Müller cells at the vitreous body and in those parts of the cells that contact retinal blood vessels. The data presented here suggest that laminins containing the β2 and γ3 chains are critical for scaffolding the channels mainly at the ILM. It has been described that β-dystroglycan was still present at retinal blood vessels in β2^−/−^/γ3^−/−^ mice [34]. This is in agreement with our finding that immunoreactivities for laminin (Fig. 1D′) and aquaporin-4 (Fig. 1E′) were still found around retinal vessels in knockout mice. Laminin α1β1γ1, the α2 chain (component of laminin α2β1γ1), and the α5 chain (component of laminin α5β1γ1) are expressed at vascular sites in rat and human retinae [26]. Laminin α1β1γ1 is known to interact with α-dystroglycan in Müller cell cultures [35]. Moreover, it has been shown that laminin α1β1γ1 can modulate clustering of dystroglycan and Kir4.1 in these cells [47]. A similar mechanism has been found for aquaporin-4 in brain astrocytes [48]. Laminin α1β1γ1 localized in the vascular basement membrane may therefore contribute to the scaffolding of the remaining aquaporin-4 found at this location in β2^−/−^/γ3^−/−^ mice. Moreover, we found a downregulation of Kir4.1 mRNA and of Kir4.1 and aquaporin-4 proteins suggesting that β2 and γ3 containing laminins also have an impact on expression levels of these membrane proteins as well as to stabilize their subcellular localization. Because we did not find a significant reduction of aquaporin-4 mRNA (Fig. 3), it can be speculated that the lack of β2 and γ3 laminins affects aquaporin-4 expression not at the transcriptional level but rather by reducing its stabilization in the membrane resulting in decreased incorporation in the membrane and increased protein turnover.

As shown by immunocytochemistry, the Müller cell endfoot is the compartment most dramatically affected by the downregulation in both proteins. This result is not surprising because in the wild-type retina, the Müller cell endfeet directly contact the laminin-containing ILM. Moreover, β-dystroglycan expression at vascular basement membrane is intact in β2^−/−^/γ3^−/−^ mice [34]. Our data suggest a key role of β2 and γ3 laminin chains in the ILM for the expression of both Kir4.1 and aquaporin-4 and, thus, for the maintenance of retinal potassium and water homeostasis. Hence, our data support the hypothesis that laminins α4β2γ3 and α5β2γ3 are critical for the generation of Müller cell polarity [34].

The reduction in expression levels is most likely due to alterations in signalling via the DAP complex, due to the lack of β2 and γ3 laminin subunits. These findings are consistent with data from Müller cells of the dystrophin Dp71 knockout mice [36]. Dp71 is another member of the DAP complex. Knockout of Dp71 resulted in a decrease of aquaporin-4 protein and a redistribution of Kir4.1 from endfeet to soma and distal processes, accompanied by an alteration of its physiological activity. The knockout of Dp71 is partly compensated by an upregulation of utrophin, also a member of the DAP complex [36]. However, the knockout of another DAP, α-syntrophin, caused a partial loss of aquaporin-4 but no decrease of Kir4.1 in Müller cell endfeet [49]. The fact that laminins are more upstream in the signalling cascade than dystrophin could explain why we observed a downregulation of Kir4.1 and aquaporin-4 protein expression in β2^−/−^/γ3^−/−^ instead of a mere redistribution and/or disturbances in membrane insertion. In addition, compensatory mechanisms can exist between dystrophins and utrophins whereas, in the present case, the disruption of the ILM limits any possibility of compensation to other laminins normally expressed in the ILM. The fracturing of the ILM results in dramatic morphological changes in Müller cells. Most notably is the bending of Müller cells to maintain the contact with matrix molecules or detaching from the vitreoretinal interface. The disruption of the ILM induces a gliosis similar to the one observed in case of retinal detachment after enzymatic digestion of ILM components [50], [51].

Our immunohistochemical and qPCR data suggest a downregulation of Kir4.1 channels. These results could be verified in patch-clamp experiments. Kir-mediated inward currents were significantly smaller in the β2^−/−^/γ3^−/−^ mice as compared to three weeks old wild-type animals. Because the dominant Kir conductance determines the very negative membrane potential of Müller cells, the small current amplitudes are accompanied by less negative membrane potentials. We observed that the lack of β2 and γ3 laminins causes an abnormal maturation of Müller cells. The current patterns shown in Figure 4A are similar to those recorded in radial glial cells of wild-type mice at the age of P8 to P13 [40]. Because no developmental alteration was observed between 3 and 4 weeks of age, it seems to be likely that the postnatal development of Müller cells from β2^−/−^/γ3^−/−^ mice is aborted at an early stage. This is in line with the described alterations of retinal histogenesis in this mouse model which were referred to as dysplasia, i.e., disturbances in development causing an abnormal tissue structure [34]. The observed increase in the membrane capacitance might be caused by a hypertrophy of Müller cells which would point to a gliotic response, also indicated by the increase in GFAP mRNA and protein. Mice older than P32 could not be analysed because this was the oldest age reached by a β2^−/−^/γ3^−/−^ mouse.

The data on Kir4.1 localization and function in β2^−/−^/γ3^−/−^ mice are of special interest because a number of recent articles demonstrated the involvement of mutations of the *KCNJ10* gene in human disorders, such as the EAST syndrome [14]–[18]. Interestingly, one of the respective patients displayed a mutation deleting the PDZ-binding domain of the channel. This domain is known to be required for clustering of Kir4.1 at the cell surface [46], [47]. Consequently, the mutation prevented the correct localization of the channel in the cell membrane [14]. It has been verified in cell cultures that this mutation results in a complete lack of membrane currents [15].

Taken together, these results confirm that functional expression of laminin subunits β2 and γ3 and, therefore, a functional formation of the ILM are necessary for the highly asymmetric and functional expression of Kir4.1 and aquaporin-4 in Müller cells.
